# The Races of Men: A Fragment

**Published:** 1851-01

**Authors:** 


Art. VII.—*The Races of Men:* A Fragment. By Robert Knox, M. D. Lea & Blanchard. Philadelphia. 1850.

There has never been a time, when the subject of the races of men attracted such universal attention as it is commanding at present. All departments of Natural science are lending their aid to the elucidation of the subject, and its full explanation can be made alone through the medium of a number of the master interpreters in each of a large variety of the sections of Natural Science. If Philology alone were equal to the work, we might have hoped much from the labors of William Humboldt; if profound acquaintance with Paleontology and all the minute details of Geology could have accomplished the great desideratum, what might not have been hoped from the labors of Agassiz, and if a thorough mastery of Natural history could untie the Gordian Knot, we might turn to Dr. Bachman with confident hope. But alas, none of these minds have unveiled the deep mysteries of the great subject, and we hope, that we may be pardoned for saying that the author before us has scarcely any qualifications for the main question he has undertaken to discuss.

But we do not intend, at present, to enter into a disquisition on Ethnology, because a mere statement of the elementary principles of the subject would encroach so much upon the limited space allotted to practical medicine, that we cannot now say much on the matter of the work of Dr. Knox. But we shall take an early occasion to do so, for we regard this question of Races, as one eminently adapted to the study of medical men, and we cannot refrain from expressing our thanks to our contemporary of the *Charleston Medical Journal* for the large and well filled space, given to some of the branches of Ethnology, during the past few months in the pages of that Journal.

Dr. Knox has entitled his work a fragment, and it is a true description. His knowledge of the subject matter seems to be very fragmentary, although he has managed portions of it with a great deal of skill. But we must stop for the present.

These works may be obtained at the book store of Beckwith & Morton.

